# The Relationship of Omental and Subcutaneous Adipocyte Size to Metabolic Disease in Severe Obesity

**DOI:** 10.1371/journal.pone.0009997

**Published:** 2010-04-01

**Authors:** Jean O'Connell, Lydia Lynch, Tom J. Cawood, Anna Kwasnik, Niamh Nolan, Justin Geoghegan, Aiden McCormick, Cliona O'Farrelly, Donal O'Shea

**Affiliations:** 1 Obesity Research Group, Education and Research Centre, St Vincent's University Hospital, Dublin, Ireland; 2 Department of Endocrinology, Christchurch Hospital, Christchurch, New Zealand; 3 Department of Pathology, St Vincent's University Hospital and St Columcille's Hospital, Dublin, Ireland; 4 Department of Surgery, St Vincent's University Hospital and St Columcille's Hospital, Dublin, Ireland; 5 Liver Transplant Unit, St Vincent's University Hospital, Dublin, Ireland; 6 School of Biochemistry and Immunology, Trinity College, Dublin, Ireland; 7 Department of Endocrinology, St Vincent's University Hospital and St Columcille's Hospital, Dublin, Ireland; Institute of Preventive Medicine, Denmark

## Abstract

**Objective:**

Several studies have reported the existence of a subgroup of obese individuals with normal metabolic profiles. It remains unclear what factors are responsible for this phenomenon. We proposed that adipocyte size might be a key factor in the protection of metabolically healthy obese (MHO) individuals from the adverse effects of obesity.

**Subjects:**

Thirty-five patients undergoing bariatric surgery were classified as MHO (n = 15) or metabolically unhealthy obese (MUO, n = 20) according to cut-off points adapted from the International Diabetes Federation definition of the metabolic syndrome. Median body mass index (BMI) was 48 (range 40–71).

**Results:**

There was a moderate correlation between omental adipocyte size and subcutaneous adipocyte size (r = 0.59, p<0.05). The MHO group had significantly lower mean omental adipocyte size (80.9±10.9 µm) when compared with metabolically unhealthy patients (100.0±7.6 µm, p<0.0001). Mean subcutaneous adipocyte size was similar between the two groups (104.1±8.5 µm versus 107.9±7.1 µm). Omental, but not subcutaneous adipocyte size, correlated with the degree of insulin resistance as measured by HOMA-IR (r = 0.73, p<0.0005), as well as other metabolic parameters including triglyceride/HDL-cholesterol ratio and HbA1c. Twenty-eight patients consented to liver biopsy. Of these, 46% had steatohepatitis and fibrosis. Fifty percent (including all the MHO patients) had steatosis only. Both omental and subcutaneous adipocyte size were significantly associated with the degree of steatosis (r = 0.66, p<0.0001 and r = 0.63, p<0.005 respectively). However, only omental adipocyte size was an independent predictor of the presence or absence of fibrosis.

**Conclusion:**

Metabolically healthy individuals are a distinct subgroup of the severely obese. Both subcutaneous and omental adipocyte size correlated positively with the degree of fatty liver, but only omental adipocyte size was related to metabolic health, and possibly progression from hepatic steatosis to fibrosis.

## Introduction

The prevalence of chronic conditions such as type 2 diabetes, hypertension and nonalcoholic fatty liver disease (NAFLD) increases with increasing weight. However, not all obese individuals display the expected features of metabolic dysfunction. Up to 30% of obese individuals are metabolically healthy, and therefore may be protected from the increased morbidity and mortality associated with excess weight[1], [2], [3]. Several papers support the concept of metabolically healthy obese (MHO) individuals and investigate what factors characterize this phenotype[3], [4], [5], [6]. Stefan et al reported that individuals with ‘benign obesity’ demonstrated a higher degree of insulin sensitivity, lower levels of ectopic fat in liver and skeletal muscle, as well as lower carotid artery intima-media thickness, when compared with an insulin resistant obese group. However, waist circumference and degree of visceral adiposity were similar between the two groups[5].

Adipose tissue is now firmly established as an endocrine organ, producing a variety of important steroids, cytokines and adipokines[7], [8]. The adipocyte is therefore an obvious potential determinant of the local and systemic metabolic environment. Over 4 decades ago, adipocyte size was shown to vary inversely with adipocyte insulin sensitivity[9]. More recently, studies have shown functional differences in large and small adipocytes from the same subjects, including altered gene expression profiles[10] and a blunting of GLUT-4 translocation in response to insulin stimulation in larger adipocytes[11]. Adipocyte size has also been shown to influence adipokine secretion, with increasing adipocyte size resulting in a shift towards dominance of pro-inflammatory adipokines[12].

In general, metabolic disorders are associated more strongly with visceral adiposity, rather than with subcutaneous adiposity. Depot-related differences exist in adipocyte responses to lipolytic and lipogenic stimuli, in adipocyte apoptosis, expression of adipokine receptors, and secretion of adipokines[13], [14], [15], [16]. Also, the anatomic location of visceral adipose tissue means that fatty acids are released directly into the portal circulation and fat accumulation in the liver has been shown to be an important feature of the metabolic syndrome[17], [18].

Few studies have focused on individuals with a body mass index (BMI) >40, the fastest growing category of obesity[19]. The aim of this study therefore, was to determine if adipocyte size is one of the factors associated with the MHO phenotype in severe obesity. We also investigated the relationship between adipocyte size and the degree of NAFLD in these subjects.

## Methods

### Ethics statement

St Vincent's University Hospital Ethics Committee approved this study. Written informed consent was obtained from every participant prior to the start of any research activities.

### Subjects

We studied 48 consecutive, severely obese patients undergoing bariatric surgery. All patients were attending the weight management service for at least 1 year prior to surgery. They were weighed monthly during this time. Patients were excluded from the study if they had significant weight loss in the preceding 6 months *(n = 4)*, or if they were taking medications that could affect metabolic and/or hepatic parameters *(n = 9)*. We obtained blood and omental adipose tissue samples from the remaining 35 subjects. Nineteen of the 35 subjects consented to subcutaneous adipose tissue sampling. With regard to age, BMI, and metabolic parameters, there was no significant difference between the group who consented to biopsy and those who did not. Mean age was 42±7 years, median weight was 150 kgs (range 103–240), and median BMI was 48 kgm^−2^ (range 40–71). There were 10 males and 25 females. Fifteen patients were classified as metabolically healthy obese, based on their metabolic profile, as detailed below. Six of the remaining 20 unhealthy patients had type 2 diabetes (DM2).

### Defining metabolically healthy obese subjects

Metabolically healthy obese (MHO) subjects had no history of cardiovascular, respiratory or metabolic diseases. They were not on any lipid-lowering, anti-hypertensive, or hypoglycemic agents. Clinical examination was unremarkable and thyroid status was normal. Fasting glucose level was ≤5.6 mmol/L, blood pressure was ≤135/85, and TGL/HDL cholesterol ratio was ≤1.65 (men) or ≤1.32 (women). These cut-off points were adapted from the International Diabetes Federation worldwide consensus definition of the metabolic syndrome, 2006. The plasma triglyceride/high-density lipoprotein cholesterol concentration ratio was used as this has been shown to provide a simple means of identifying insulin-resistant, dyslipidemic patients who are likely to be at increased risk of cardiovascular disease[20]. Metabolically unhealthy obese (MUO) subjects were defined by failure to meet at least one of the criteria above.

### Adipose tissue samples and determination of adipocyte size

Approximately 10–30 gms of omental adipose tissue, or 5–10 gms of abdominal subcutaneous adipose tissue, was obtained at the time of bariatric surgery. A piece of this tissue was immediately fixed in formalin, prior to paraffin mounting and preparation of H&E slides. The remaining tissue was placed in warm DMEM-F12 medium, supplemented with 10%FCS, transported to the lab, and processed within 1 hour. Adipocyte size was assessed by 2 methods.

Method I *(n = 35)*: Digital photomicrographs of H&E slides were analyzed using UTHSCA Image Tool Software (University of Texas Health Science Center). Two individual operators calculated the maximal diameter of 100 adjacent adipocytes from each of 4 separate photographs. This data was transferred to an Excel program to calculate the mean adipocyte diameter and standard deviation for each sample. Both operators were blinded to patients' clinical details.

Method II *(n = 10)*: Fresh samples of adipose tissue were incubated in collagenase and DMEM-F12 medium (1 mg/ml collagenase type II (Sigma C-6885)) for 60 minutes in a metabolic shaker (60–80 strokes/min) at 37°C. The cell suspension was filtered through a 250 µm Nitex mesh and washed 3 times in warm medium - cells were allowed to float by gravity and the infranatant removed using a syringe and 18 g needle, prior to adding fresh medium, gently inverting the container each time to ensure an even suspension. A 50 µL aliquot of the cell fraction (suspended in medium) was diluted with 100 µL medium and 50 µL trypan blue (0.4% in H_2_O). Ten µL of this solution was transferred to a Neubauer chamber, digital photographs taken, and measurements of mean adipocyte diameter calculated as above.

In order to ensure reproducibility of both methods, 2 operators performed the analysis twice. The inter-individual correlation was calculated to compare the results obtained by both individuals. The intra-individual correlation was calculated to compare the results obtained by the same individual repeating the measurements twice.

### Liver biopsies and determination of the degree of fatty liver

Liver biopsies were performed by the surgeon peri-operatively, using a ‘Tru-Cut’ needle and immediately fixed in formalin. Biopsy fragments were at least 10 mm, and contained at least 8 portal triads, in order to ensure accurate histological assessment. Liver tissue was paraffin-embedded, stained with hematoxylin-eosin, and with a standard panel of special stains (PAS +/− diastase, iron, trichrome, reticulin and shikata).

The sections were then examined by a pathologist blinded to the clinical details and the type and degree of steatosis reported. An eyepiece graticule with a 100-point grid was inserted in the eyepiece and the biopsy was viewed using the 10x objective. The number of hits on fatty hepatocytes and normal hepatocytes was counted; the process was repeated four times in each case. The results are given as the percentage of biopsy area with fat deposition. A diagnosis of steatohepatitis was made if there was ballooning degeneration with Mallory's hyaline, neutrophilic infiltration and perisinusoidal or pericellular fibrosis[21]. Fibrosis was assessed with the aid of the connective tissue stains trichrome and shikata and classified as to its anatomical distribution and stage: perisinusoidal and pericellular fibrosis, portal, periportal fibrosis, septal, bridging fibrosis or cirrhosis.

### Statistical Analyses

Data in the text and tables are presented as mean +/− standard deviation, or median, with the range in parentheses, as appropriate. Student's unpaired t-test or Mann-Whitney U tests were used to test for differences between healthy and unhealthy groups, and groups with and without hepatic fibrosis, as appropriate. One-way analysis of variance (ANOVA) was used to test for differences between healthy, unhealthy and DM2 groups. Post-hoc comparisons were assessed by Tukey HSD test.

Relationships between clinical and metabolic parameters and adipocyte size were visualized by scatter plots and assessed by Spearman rank correlation test (Spearman's rho or r). Overall correlation results were confirmed by subset correlation analysis of the MHO and MUO groups to ensure that arrangement of the groups did not artificially increase the correlation coefficient. Scatter plots did not demonstrate distinct data groups. Direct logistic regression was performed to assess the impact of a number of factors on the likelihood of hepatic fibrosis.

P values <0.05 were considered to be statistically significant. When multiple analyses were performed on data sets, a Bonferroni correction was used to set a higher alpha level.

## Results

### Measurement of adipocyte size

**Figure 1:**
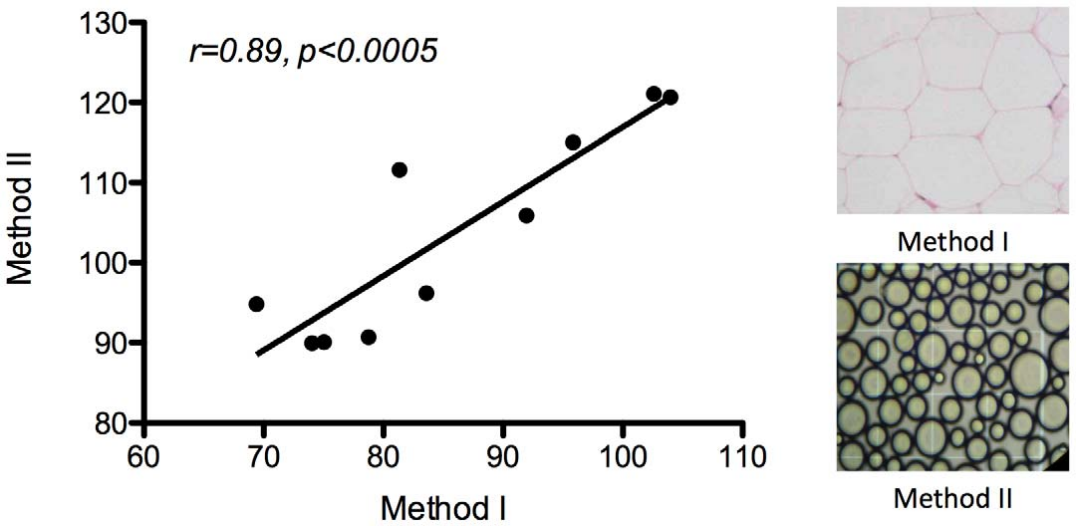
Adipocyte size results (micrometers) for Method I and Method II.

There was good agreement between methods I and II (Figure 1), although the average cell diameter determined by Method I was smaller than that obtained by Method II by a factor of approximately 1.2. Intra-individual and inter-individual results had correlation coefficients of 0.92 and 0.89, respectively. Further analyses used measurements obtained by Method I.

### Metabolic profile of healthy and unhealthy groups

Table 1 summarizes the clinical features of the MHO and the MUO group. A smaller group with type 2 diabetes (DM2) was considered separately to the MUO group. This group was slightly older than the other groups, and predominantly female. The MHO and MUO groups were matched for age, BMI, and gender ratio. By design, the groups were significantly different with regard to blood pressure, fasting blood glucose levels, and lipid profile.

**Table 1:** Metabolically healthy obese (MHO) compared to metabolically unhealthy obese (MUO) and metabolically unhealthy obese with type 2 diabetes (DM2).

	MHO (n = 15)	MUO (n = 14)	DM2 (n = 6)	p value
**Anthropometric and metabolic profile**				
M ♂ / F ♀	5/10	4/10	1/5	
Age	40 (23–52)	42 (33–56)	46 (38–53)	ns
BMI (kgs/m2)	48 (40–59)	51 (44–71)	50 (40–55)	ns
Systolic BP (mmHg)	119 (100–135)	132 (105–160)	137 (130–172)	<0.05
Diastolic BP (mmHg)	76 (50–85)	81 (62–105)	83 (70–95)	ns
Fasting Glucose (mmol/L)	4.9 (4.2–5.4)	5.3 (4.1–6.0)	7.6 (7.2–12.2)	<0.0005
HbA1c (%)	5.4 (4.8–5.6)	5.6 (5.2–6.2)	7.4 (5.1–10.1)	<0.05
Cholesterol (mmol/L)	4.9 (3.1–5.7)	5.7 (4–7.2)	4.9 (4–8.1)	ns
Triglyceride (mmol/L)	1.2 (0.7–1.7)	1.9 (1.3–3.2)	2.1 (1.6–6.1)	<0.0005
HDL (mmol/L)	1.3 (0.9–1.8)	1.2 (0.8–1.5)	0.9 (0.8–1.7)	ns
TGL/HDL ratio	0.9 (0.5–1.5)	1.6 (1.0–3.7)	2.5 (0.9–5.8)	<0.0005
Fasting Insulin (µU/mL)	13.8 (6.7–17)	24.8 (12.9–42.2)		<0.05
HOMA-IR	1.7 (0.9–2.0)	3.0 (1.6–5.6)		<0.05
**Adipocyte size (µm)**				
Omental *n = 35* 10 ♂ - 92.5 (16.6) 25 ♀ - 91.6 (11.9)	80.9 (10.9)	98.2 (10.2)	104.3 (4.2)	<0.0001
Subcutaneous *n = 19* 6 ♂ - 110.7 (4.2) 13 ♀ - 104.4 (7.9)	104.1 (8.5) (3 ♂, 4 ♀)	104.9 (5.0) (2 ♂, 6 ♀)	113.2 (6.9) (1 ♂, 3 ♀)	ns
**Liver biopsy results**				
Normal or steatosis only	9 of 9	5 of 14	1 of 5	
NASH and/or fibrosis	None	9 of 14	4 of 5	
Degree of steatosis (%)	3 (0–56)	47 (3–70)	74 (32–98)	<0.005

Values are expressed as median and range in parentheses, except for adipocyte size, expressed as mean +/− standard deviation in parentheses.

### Adipocyte size and metabolic status

Subcutaneous adipocytes were always larger than omental adipocytes, with a moderate association seen between paired samples (r = 0.59, p<0.01). Mean omental adipocyte size was 91.8±13.2 µm, mean subcutaneous adipocyte size was 106.4±7.6 µm. Gender did not appear to influence mean omental adipocyte size (male: 92.5±16.6 µm, female: 91.6±11.9 µm; p = 0.81), however subcutaneous adipocyte size showed a trend towards a higher mean value in men (male: 110.7±4.2 µm, female: 104.4±7.9 µm; p = 0.07). Neither omental nor subcutaneous adipocyte size showed any correlation with age or body mass index.

**Figure 2:**
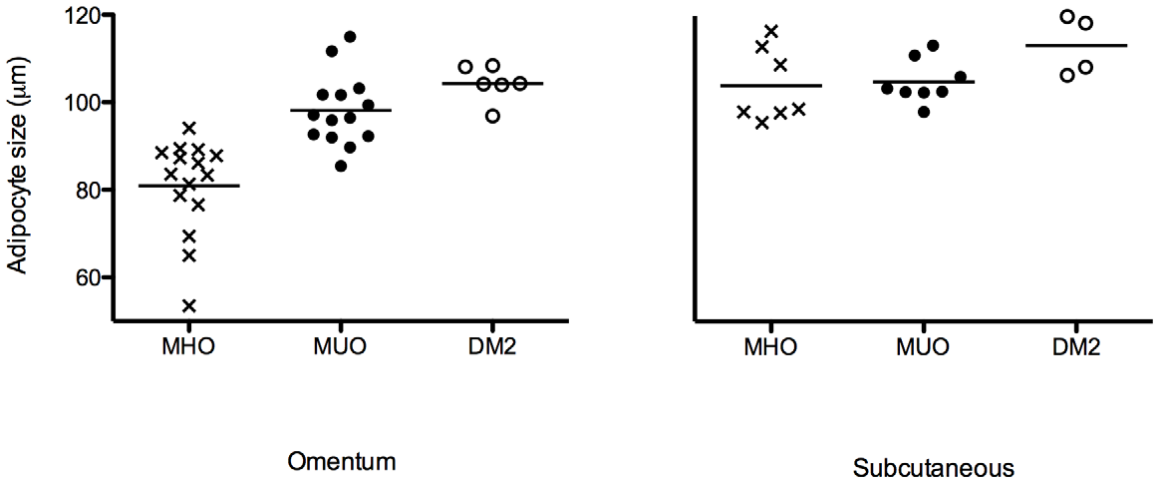
Adipocyte size in obese subjects grouped by metabolic profile. Metabolically healthy obese  =  MHO, metabolically unhealthy obese  =  MUO, metabolically unhealthy obese patients with Type 2 Diabetes  =  DM2. Mean omental adipocyte diameter (represented by the horizontal lines) was 80.9±10.9 µm in MHO, 98.2±10.2 µm in MUO, and 104.3+/−4.2 µm in DM2. Mean subcutaneous diameter was 104.1±8.5 µm in MHO, 104.9±5.0 µm in MUO, and 113.2±6.9 µm in DM2.

The MHO group had a significantly lower mean omental adipocyte size (80.9±10.9 µm) when compared with the MUO group (100.0±7.6 µm, p<0.0001). The unhealthy patients with DM2 appeared to have a slightly larger adipocyte size when compared to the unhealthy group without DM2, but this did not reach statistical significance (104.3±4.2 µm and 98.2±8.2 respectively, p = 0.10). Subcutaneous adipocyte size was similar in both healthy and unhealthy groups. (Figure 2)

**Figure 3:**
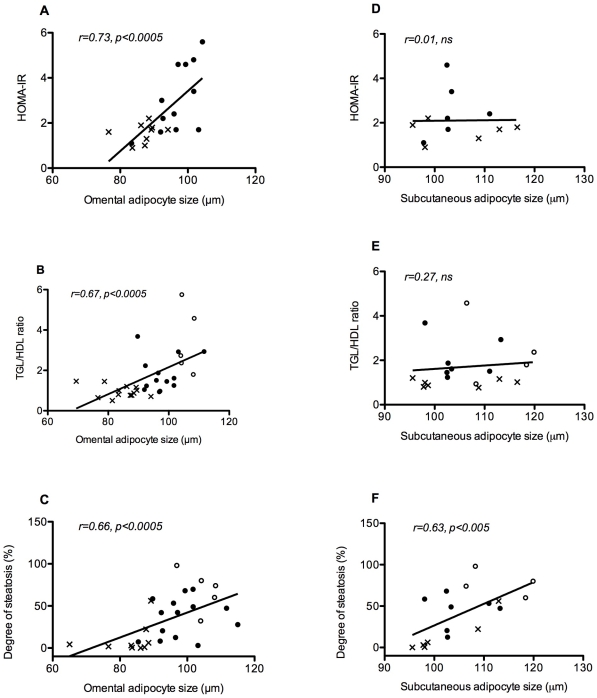
Correlations of adipocyte size with metabolic parameters. Omental adipocyte size (A, B and C) and subcutaneous adipocyte size (D, E and F) correlated with the degree of insulin resistance as measured by HOMA-IR, the TGL/HDL ratio, and the degree of hepatic steatosis. X  =  MHO, • =  MUO, ○ =  DM2

Omental adipocyte size was shown to correlate strongly with the degree of insulin resistance as measured by HOMA-IR (r = 0.73, p<0.0005), as well as other metabolic parameters, particularly triglyceride level (r = 0.65, p<0.0005), TGL/HDL ratio (r = 0.67, p<0.0005) and HbA1c (r = 0.50, p<0.005). Subcutaneous adipocyte size showed no correlation with any of these metabolic parameters. (Figure 3)

Body mass index was associated with systolic blood pressure (r = 0.54, p<0.005), but there was no association with other metabolic parameters.

### Nonalcoholic fatty liver disease (NAFLD) in patients with BMI>40

Twenty-eight of the 35 patients consented to an intra-operative liver biopsy. Of these 28 biopsies, one biopsy was classified as normal (from a MHO subject). Fourteen subjects (50%) had evidence of steatosis only. Thirteen subjects (46%) had evidence of nonalcoholic steatohepatitis (NASH) and varying degrees of fibrosis. None of the MHO patients had evidence of NASH or fibrosis.

The degree of steatosis present showed a linear trend across metabolic groups. The MHO group had the lowest median degree of steatosis [3 (0–56%)]. The MUO group without DM2 had an intermediate degree of steatosis [47.3 (3–70%)], between the MHO and the group with DM2 [74 (32–98%)]; p<0.005). (Table 1)

### Adipocyte size and NAFLD

Omental and subcutaneous adipocyte size showed strong correlations with the degree of hepatic steatosis (omental: r = 0.66, p<0.0005, subcutaneous: r = 0.63, p<0.05). (Figure 3)

**Figure 4:**
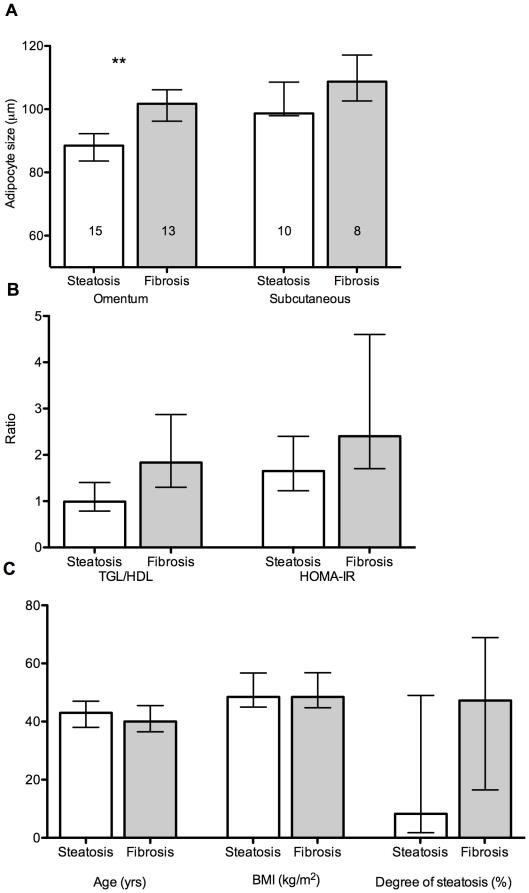
Omental adipocyte size, metabolic parameters and hepatic fibrosis. A) Omental and subcutaneous adipocyte size, B) TGL/HDL and HOMA-IR ratios and C) Age, BMI and degree of steatosis, in subjects with hepatic steatosis alone, compared with subjects with evidence of hepatic fibrosis (** p<0.005). The data are presented as median values and inter-quartile range.

The mean adipocyte size of the patients with evidence of hepatic fibrosis was 100.6±7.2 µm (om) and 109.6.0±7.2 µm (sc), significantly larger than the mean adipocyte size of patients with normal/fatty livers only [89.0±11.1 µm (om) and 102.4±6.2 µm (sc); p<0.005 (om), p<0.05 (sc)] as seen in Figure 4.

Age, body mass index, blood pressure and liver function tests did not differ significantly between the two groups. With regard to metabolic parameters, the group with hepatic fibrosis had trends toward higher median TGL/HDL ratio, higher median HOMA-IR, as well as a higher median degree of steatosis, when compared with the normal/fatty liver only group (Figure 4). However, after adjustment for multiple analyses, omental adipocyte size was the only parameter that remained significantly higher in the group with hepatic fibrosis.

Direct logistic regression was performed to assess the impact of a number of factors on the likelihood of hepatic fibrosis being present. The model included the independent variables age, sex, BMI, degree of steatosis, omental adipocyte size, triglyceride to HDL-cholesterol ratio (TGL/HDL-C) and fasting glucose [χ^2^ (7, n = 46) = 20.4, p<0.005]. Only adipocyte size made a unique statistically significant contribution to the model.

## Discussion

We have shown that severely obese patients with healthy metabolic profiles have significantly smaller omental adipocytes than equally obese patients with unhealthy metabolic profiles. Furthermore, omental adipocyte size strongly correlated with the degree of insulin resistance and the degree of hepatic steatosis. Obese individuals with smaller adipocytes had no evidence of hepatic fibrosis. Subcutaneous adipocyte size was significantly associated with the degree of fat in the liver, but had no association with metabolic parameters, and did not predict presence or absence of hepatic fibrosis. Body mass index was not associated with adipocyte size, and was also a poor indicator of metabolic health or degree of fatty liver disease in this patient group (median BMI 48).

Other studies have investigated the factors responsible for protecting MHO individuals from developing co-morbidities such as diabetes and dyslipidemia[4], [5], [6]. Body fat distribution is thought to play a role in influencing the metabolic environment. One study reported that lower amounts of visceral adipose tissue explained 22% of the variance in insulin sensitivity between MHO and MUO groups[4]. However, it has also been shown that while the amount of visceral fat is a strong predictor of insulin resistance in normal or overweight individuals, the predictive effect of visceral fat mass is relatively weak in obese patients[5]. Obese patients all have a significant degree of visceral adiposity. In this patient group, the size of the omental adipocytes may be more important than the size of the omental fat depot.

The overflow hypothesis proposes that as the size of an adipocyte increases, it will eventually reach a limit and be unable to store further lipid. Excess fatty acids then ‘overflow’ to ectopic sites, including muscle and liver, leading to peripheral and hepatic insulin resistance, respectively[22], [23]. The results of our study support this theory, suggesting that lipid overflow from hypertrophied subcutaneous fat cells accumulates in the omentum and liver. Subsequent omental fat cell hypertrophy leads to more detrimental metabolic effects, possibly because of close proximity to hepatic and visceral immune cell populations.

In vitro work has shown that increasing adipocyte size varies inversely with adipocyte insulin sensitivity[9]. Large adipocytes are more likely to produce pro-inflammatory adipokines[12] and to demonstrate increased basal and catecholamine-stimulated lipolysis[24]. Large adipocytes also have altered gene expression profiles when compared with smaller adipocytes[10]. These studies have focused on subcutaneous adipocytes. However, increased production of pro-inflammatory cytokines and other adverse qualities of fat cells in vitro, may not translate to adverse outcomes in vivo. Our study suggests that adipocyte size, in severely obese individuals, is a strong indicator of metabolic health, particularly when studying adipocytes from the omental depot.

Subcutaneous adipocytes may not have a direct impact on metabolic dysfunction, but they still may play a key role as the initiating factor in the process of fat overflow to ectopic sites. Lonn M et al showed that abdominal subcutaneous adipocyte size predicted the onset of DM2 independently of body fat percentage or waist to hip ratio in a Swedish cohort of women[25]. This work confirmed the original finding by Weyer C et al in Pima Indians[26]. The primary defect may be an inability of subcutaneous adipose tissue proliferation and differentiation[27], [28]. In the setting of caloric excess, it may be that some individuals have ‘healthier’ subcutaneous fat, capable of significant hyperplastic expansion, and consequently a lesser degree of fat overflow and omental adipocyte hypertrophy. The degree to which subcutaneous and omental fat can expand to accommodate excess calories may be highly variable across individuals and degrees of body fat, perhaps determined by genetic, intrauterine or environmental influences later in life.

In the setting of extremely obese individuals, as in our cohort, the subcutaneous adipocytes may all have reached their maximum expansion limit, and therefore MHO and MUO have similar mean adipocyte size. However, MHO may have increased pre-adipocyte and adipocyte number, and therefore overall greater subcutaneous storage capacity. This would explain the smaller omental adipocyte size, and consequent metabolic benefits, seen in this group. Arner et al recently showed that total subcutaneous adipocyte number was greatest in pronounced hyperplasia and smallest in pronounced hypertrophy[29]. The mean BMI in this study was 33 kg/m^2^ so this association may be blunted at more extreme levels of obesity (the mean BMI of our cohort was 50 kg/m^2^).

We have studied abdominal subcutaneous fat in severely obese subjects. Previous work has demonstrated morphological and metabolic differences between abdominal, gluteal and femoral subcutaneous adipose tissue depots[30], [31], [32]. However, increasing body fat can also influence these differences. In one such study, adipocyte size was shown to vary across the abdominal, gluteal and femoral depots at normal and overweight BMI's. Obese individuals had similar adipocyte size at all 3 depots[31]. Lower body subcutaneous fat may have a beneficial influence on metabolic health but this influence may vary between normal weight, overweight and obese individuals. Further research, comparing the phenotypic and metabolic features of different subcutaneous fat depots in metabolically healthy and unhealthy obese individuals, would help to clarify the importance of subcutaneous fat in metabolic disease. Intra-peritoneal visceral fat can also be subdivided into omental and mesenteric fat[33], with emerging evidence that mesenteric adipose tissue has a distinct role in the insulin resistance of diabetes and the metabolic syndrome[34], [35], [36].

Our study focuses on subjects with a median BMI of 48 kg/m^2^ (mean BMI 50), a group lying at the extreme end of the obesity spectrum. The associations between subcutaneous and omental adipocyte size and metabolic parameters may vary in normal or overweight individuals. This could account for the differences between our findings, and those of other studies reporting associations between subcutaneous adipocyte size and metabolic risk, where the mean BMI of populations studied is usually 25–30 kg/m^2^ [31], [37]. Another limitation of our study is that only nineteen of the 35 subjects agreed to a subcutaneous fat biopsy. However, correlation analysis of these 19 subjects alone (who all underwent liver biopsy) confirms the results reported for the whole group. Also, if we had greater numbers in our study, it would have been preferable to analyse males and females separately. In order to minimize gender influences we aimed to have a similar gender ratio across the different groups (MHO and MUO have similar gender ratios, DM2 has more females). Differences in fat cell size between males and females have been shown to be more prominent in peripheral subcutaneous fat sites[30]. The relationship between fat cell size and lipoprotein lipase activity is similar in males and females in abdominal fat but not gluteal fat[30]. Lastly, catecholamine mediated leg free fatty acid release is lower in women than in men, whereas free fatty acid release from the upper body depots is comparable[38]. In summary, gender-related differences appear to be less pronounced in abdominal subcutaneous fat depots.

NAFLD has been described as an additional feature of the metabolic syndrome and there is a strong link between NAFLD and obesity. However, similar to other parameters of metabolic health, not all obese individuals develop NAFLD. In the setting of severe obesity, the prevalence of NAFLD ranges from 75–85%[39]. Within our cohort, 96% of patients had NAFLD and, 48% of those patients had evidence of steatohepatitis and fibrosis.

Predicting which patient will progress from steatosis to fibrosis is difficult, even with information provided by liver biopsy. Recent studies indicate that the histological severity of NAFLD correlates with the indices of the metabolic syndrome, particularly degree of insulin resistance[40], [41]. We have shown that obese individuals with larger adipocytes are more likely to develop steatosis. Those with larger omental adipocytes are more likely to progress to hepatic fibrosis. This may be secondary to the association of increased adipocyte size and insulin resistance. However, larger adipocytes are positively correlated with adipose tissue macrophage number and production of pro-inflammatory cytokines TNF-α and IL-6 in mice[42]. Omental macrophage accumulation is also associated with the severity of fibroinflammatory liver damage, independently of degree of insulin resistance[43], [44]. In addition, adipocyte volume was found to have a significant positive correlation with serum TNF-α levels and soluble TNF receptors in lean and overweight patients[45]. Finally, secretion of the pro-inflammatory adipocytokines leptin, IL-6, IL-8, and monocyte chemoattractant protein-1 from cultured adipocytes correlated positively with cell size[12]. It may be that increased omental adipocyte size is also playing a key role in the ‘second hit’ of the ‘two hit’ hypothesis for steatohepatitis[46]. Increased pro-inflammatory adipocytokine production, from hypertrophied omental adipocytes and associated macrophages, may lead to a higher likelihood of progression to fibrosis from simple fatty liver.

In summary, we demonstrate a relationship between omental and subcutaneous adipocyte size and the degree of hepatic steatosis, in the severely obese. Omental, but not subcutaneous, adipocyte size was also associated with metabolic health and presence of hepatic fibrosis. Whether this relationship is causal, perhaps in part via the overflow hypothesis, remains to be elucidated. Our data support the concept of the metabolically healthy subgroup within the severely obese population, and suggest that the size of the individual's adipocytes is more important than the size of the individual.
